# Flexible Measurement of High-Slope Micro-Nano Structures with Tilted Wave Digital Holographic Microscopy

**DOI:** 10.3390/s23239526

**Published:** 2023-11-30

**Authors:** Xinyang Ma, Rui Xiong, Wei Wang, Xiangchao Zhang

**Affiliations:** 1 Shanghai Engineering Research Center of Ultra-Precision Optical Manufacturing, Fudan University, Shanghai 200438, China; 2 Institute of Optoelectronics, Fudan University, Shanghai 200438, China; 3 Yiwu Research Institute of Fudan University, Chengbei Road, Yiwu 322000, China

**Keywords:** optical measurement, digital holographic microscopy, high-slope structure, data fusion

## Abstract

Digital holographic microscopy is an important measurement method for micro-nano structures. However, when the structured features are of high-slopes, the interference fringes can become too dense to be recognized. Due to the Nyquist’s sampling limit, reliable wavefront restoration and phase unwrapping are not feasible. To address this problem, the interference fringes are proposed to be sparsified by tilting the reference wavefronts. A data fusion strategy including region extraction and tilt correction is developed for reconstructing the full-area surface topographies. Experimental results of high-slope elements demonstrate the validity and reliability of the proposed method.

## 1. Introduction

The three-dimensional (3D) surface topographies of micro-nano structures are of great importance for functional materials, MEMS, and micro-opto-electronics devices [1]. Nowadays, various optical metrology techniques have been developed for measuring micro-nano structures, including coherent scanning interferometry [2], confocal microscopy [3,4], digital holographic microscopy (DHM) [5], etc. The confocal microscopy is actually a focus-detection technique, with an axial resolution of tens of nanometers. Confocal microscopy utilizes pinhole spatial filtering for point-by-point illumination and scanning, with only the light returned from the focal point able to pass through the probe pinhole. By scanning the sample height-by-height, a series of sequential optical section images can be obtained. The principle of coherent scanning interferometry is based on the short coherence length of white light source. Compared to conventional phase-shift interferometers, the highest intensity contrast is only achieved when the optical path difference is 0, which is used to determine the height of the object. Due to the effect of optical diffraction caused by microstructures, the coherent scanning interferometry suffers from the batwing effect [6], which yields wavefront distortions. On the contrary, the inverse diffractive reconstruction utilized in the DHM can achieve automatic focusing and correct the wavefront distortions [7,8]. Digital holography consists of two steps: interference recording and diffraction reconstruction, which can reconstruct the complex amplitude of the object. Henceforth, DHM is suitable for measuring structured surfaces as well as diffuse surfaces with the advantages of a full field, non-contact, and high accuracy [9]. It provides a diffraction-limited lateral resolution down to a few hundred nanometers and an axial resolution of less than λ∕100 [10].

However, when the optical length difference between the object beam and the reference beam varies rapidly over the measurement area, the resulting interference fringes will become too dense or even invisible due to the limited sampling rate of the camera pixels, making the subsequent phase unwrapping infeasible. In fact, this is a common issue for interferometry [11]. Theoretically, limited by the Nyquist’s sampling principle, one surface with a local slope greater than a quarter wave per pixel [12] is not measurable by the single wavelength interferometry.

It is natural to adopt an objective lens of high magnification or a camera with a smaller pixel size to tackle this problem. However, a high magnification results in a smaller measurement field of view. And sometimes, it is not acceptable. More importantly, the improvement in fringe recognizability is limited. For example, when the microscopic objective is changed from 20× to 50×, the fringe density will be only 0.4 times the original. The improvement by reducing the camera pixel size is also limited, and the noise will increase in turn. The wavelength scanning digital holography [13] has been developed to expand the measurement range, which does not require phase unwrapping and henceforth the fringe density is no longer an issue to be taken into account. During the measurement process, the same object is measured by two light sources with wavelengths λ_1_ and λ_2_, respectively, and the measured phase profiles are noted as φ_1_ and φ_2_, respectively. The optical path difference h is obtained directly from the measured phase. However, accurate calibration is required because such an optical system responds differently to different wavelengths [13]. The sub-Nyquist interferometer [12] can improve the measurement range. The technique is based on the concept of sub-Nyquist sampling and allows the wavefront to be reconstructed from aliased fringes out to a spatial frequency equal to the MTF cutoff of the pixels. Unfortunately, it requires the use of a sparse-array sensor and the surfaces under test need to be continuously differentiable. Another solution for over-dense fringes is called null testing [14,15], which is realized by introducing an additional optical element such as a null lens or a computer-generated hologram (CGH) that fits to the nominal form of the surface under test. The compensation element must be specifically fabricated for each surface, which is not affordable in most cases. Alternatively, researchers applied adaptive optics elements such as deformable mirrors or spatial light modulators (SLMs) [16,17,18,19] as programmable wavefront compensators. Due to the unknown compensation amount, an additional monitoring system is required for deformable mirrors [20]. While the pixel pitch, filling ratio of the cell structure, and the gray-level number in SLMs directly affect the accuracy and adjustable range of wavefront control [21], which is particularly important in interferometric systems.

To solve the problem of measuring high-slope structures, we propose a flexible measuring technique by tilting the reference beam. It is straightforward to implement and unnecessary to utilize delicate adaptive optics. This paper is organized as follows. The methodology of the proposed technique is described in Section 2 and Section 3. Section 4 presents practical experiments. Finally, the paper is summarized in Section 5.

## 2. Principle of Digital Holographic Microscopy

**Figure 1:**
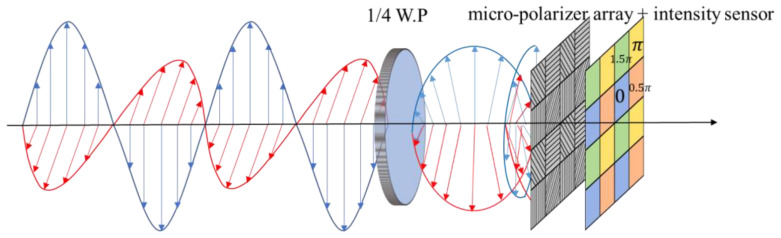
Schematic of the principle of simultaneous phase shifting.

Compared with the off-axis digital holography, the on-axis digital holography can fully utilize the Spatial-bandwidth Product (SBP) of the CCD camera [22]. However, twin-image and zero-order noise superimpose on the object image for on-axis digital holography, posing significant challenges to obtaining high-quality images. To address this issue, phase-shifting technique is required, from which the artifact-free phase and amplitude images of a sample can be reconstructed. However, conventional temporal multiple exposure phase-shifting methods is limited to static or quasi-static measurement scenarios. To overcome the limitations, simultaneous phase-shifting technique was proposed [23]. This technique enables the acquisition of several phase-shifting holograms simultaneously. An optical-path-length-shifting array device featuring a pixelated periodic thickness distribution, or a micro-polarizer array device with a pixelated periodic polarization direction distribution are usually used in simultaneous phase-shifting technique. This enables the capture of multiple digital holograms with different phase shifts in a single exposure. Here, we use the high-speed polarization camera equipped with a Sony sensor with an array of micro-polarizers. As shown in Figure 1, a large sensor array is composed of several sets of four (2 × 2) micro-polarizers, each with optical axis that span four different angles: 0, π/4 rad, π/2 rad, and 3π/4 rad. The size of an individual micro-polarizer equal to that of a single pixel on the sensor. A single sensor detects the light intensity passing through a single polarizer with a particular optical axis angle.

The principle of simultaneous phase-shifting with polarization camera is given in Figure 1. The blue line is the object light and the red line is the reference light. First, the Jones vectors of the orthogonal object and reference light are denoted as

(1) $$\left\{\begin{array}{c} O\left(x,y\right)=A_{o}\exp\left(i\varphi_{o}\right)\left[\begin{array}{c} 1\\ 0 \end{array}\right]\\ R\left(x,y\right)=A_{R}\exp\left(i\varphi_{R}\right)\left[\begin{array}{c} 0\\ 1 \end{array}\right] \end{array}\right.$$

wherein *A_O_* and *A_R_* are the amplitude of the object light and the reference light, respectively, *φ_O_* and *φ_R_* denote the phase components. Then, the light beams pass through a quarter-wave plate with its main optical axis oriented at 45° to the *x*-axis, and becomes orthogonally circularly polarized. Next, the beams pass through the polarizer attached on the sensor plane whose optical axis is *θ*, where the two orthogonal circularly polarized light beams are projected to the same direction and interfere with each other. The Jones matrices corresponding to the quarter-wave plate and the polarizer with optical axis of *θ* are *T_QW_* and *T_P_*

(2) $$T_{QW}=\frac{1+i}{2}\left[\begin{array}{cc} 1 & -i\\ -i & 1 \end{array}\right]$$

(3) $$T_{P}=[\begin{array}{cc} {cos}^{2}\theta & sin\theta cos\theta \\ sin\theta cos\theta & {sin}^{2}\theta \end{array}]$$

The Jones vector of the light beam involved in the interference are given by

(4) $$\left\{ \begin{array}{c} O^{\prime \prime }=T_{P}T_{QW}O\\ R^{\prime \prime }=T_{P}T_{QW}R \end{array}\right.$$

Finally, the light intensity at the sensor plane is expressed as

(5) $$I(x,y)={\left(O^{\prime \prime }+R^{\prime \prime }\right)}^{T}(O^{\prime \prime }+R^{\prime \prime })=\frac{1}{2}[A_{0}^{2}+A_{R}^{2}-2A_{0}A_{R}sin(\varphi -2\theta )]$$

where $\varphi =\varphi_{O}-\varphi_{R}$, which is the phase difference between the two beams. The above equation indicates that the phase shift is twice optical axis angle of the polarizer. Therefore, the four light intensities, *I*_1_, *I*_2_, *I*_3_, and *I*_4_, corresponding to the four different angles of the polarizers, *θ* = 0, π/4 rad, π/2 rad, 3π/4 rad are obtained.

**Figure 2:**
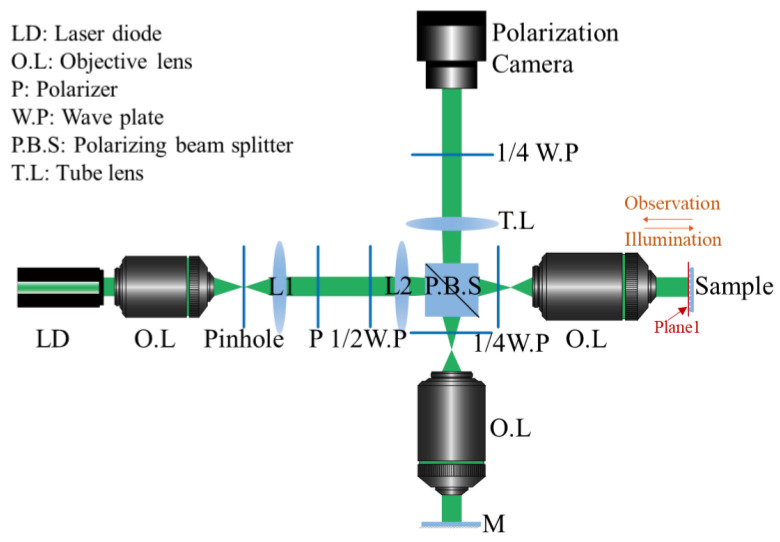
Set-up of digital holographic microscope.

With the above principles, a Linnik interferometric microscope as shown in Figure 2 is constructed. In the object arm, each object point on plane 1 can be regarded as a point source and imaged on the camera. The object and reference beams interfere and then a series of interferograms are recorded.

By use of space-division multiplexing of holograms, four phase-shifted holograms are recorded with a single image sensor with a single-shot exposure and the recorded hologram is demultiplexed into phase-shifted holograms with respect to each phase shift. However, each light intensity distribution is step sampled. The first classical approach for demosaicing is the of the super-pixel technique [24]. This method regards a group of four adjacent pixels with the different phase shift as a fundamental unit, and is straightforward to implement. However, this operation fails to account for the misalignment in the four adjacent pixels.

Specifically, these four neighboring pixels correspond to different points on the object, and because of the rapid variation in surface height on a microstructure, there are remarkable phase differences between adjacent pixels. Furthermore, it should be noted that the lateral spatial resolution of the reconstructed result will decrease, with a reduction of one-half of that of the sensor in the horizontal and vertical directions, respectively. Therefore, the light intensities in the vacant pixels should be filled using interpolation, utilizing the light intensities at the adjacent pixels. Then, the four phase-shafted holograms with the same spatial resolution as the original image are obtained by a single exposure. Taking the computational efficiency and accuracy into account, the bicubic interpolation algorithm is adopted [24].

The bicubic interpolation method estimates the interpolation result by weighted averaging the gray values of 4 × 4 known extracted pixels around the point to be interpolated, and the interpolation process can be expressed by the following equation

(6) $$I\left(x,y\right)=\overset{4}{\underset{i=1}{\sum }}\overset{4}{\underset{j=1}{\sum }}I\left(x_{i},y_{i}\right)w\left(x_{i}-x\right)w\left(y_{i}-y\right)$$

Here, *I*(*x*,*y*) denotes the value of the pixel to be interpolated and $I(x_{i},y_{i})$ is the value of the extracted pixel. The weight w is calculated according to interval between the interpolated pixel and the pixels used for interpolation. It is defined as the piecewise cubic polynomial, and we can obtain w through a frequently used approximation of a truncated Sinc function. w is expressed as

(7) $$w(x)=\left\{\begin{array}{c} \left(\alpha +2\right)\left|x{|}^{3}-\left(\alpha +3\right)\right|x{|}^{2}+1,\;0\leq |x|<1\\ \alpha \left|x{|}^{3}-5\alpha \right|x{|}^{2}+8\alpha \left|x\right|-4\alpha ,1\leq |x|<2\\ \;\;\;\;\;\;\;\;\;\;\;\;\;\;\;\;\;\;\;\;\;0\;\;\;\;\;\;\;\;\;\;\;\;\;\;\;\;\;\;\;\;\;\;\;\;\;,\;2\leq |x|\; \end{array}\right.$$

wherein the parameter *α* is adjustable and is generally set to −0.5 [25].

**Figure 3:**
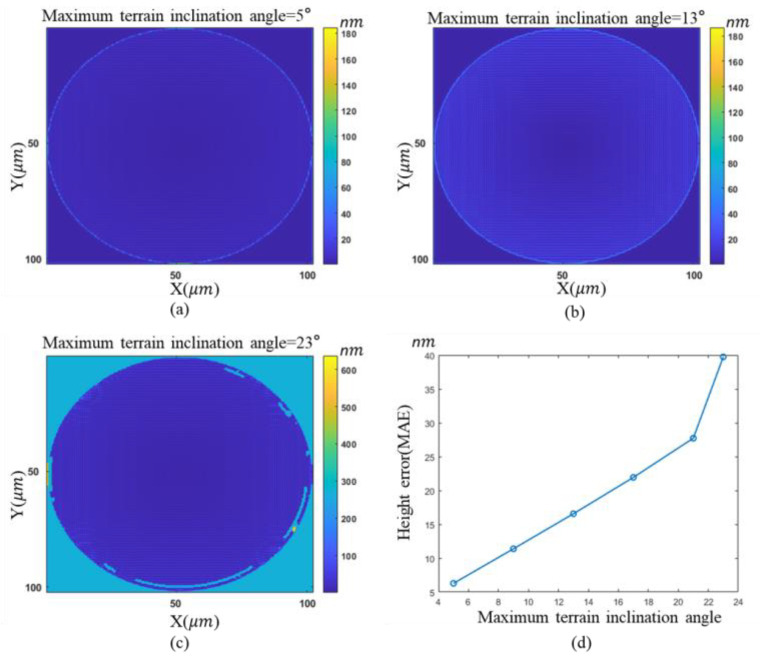
Effect of discretization on reconstruction error: (**a**–**c**) height error maps associated with different maximum terrain inclination angles; (**d**) quantitative relationship.

Then, the complex amplitude on the camera plane can be obtained using the four-step phase-shifting algorithm.

(8) $$U_{o}(x,y)=\frac{\left(I_{2}-I_{4}\right)+i\left(I_{3}-I_{1}\right)}{4A_{R}}$$

where *A*_R_ is the amplitude of the reference wave. The complex amplitude *U*_d_ of the reconstructed object wave can be obtained by autofocusing. The diffraction distance *d* which results in the greatest intensity variance after the inverse diffraction is selected [26].

(9) $$U_{d}(x,y)=F^{-1}\{F\{U_{o}(x,y)\}exp(i\frac{2\pi d}{\lambda }\sqrt{1-{\left(\lambda f_{x}\right)}^{2}-{\left(\lambda f_{y}\right)}^{2}})\}$$

where F and $F^{-1}$ denote the Fourier transform and inverse Fourier transform, respectively. Due to the limited pixel size, the interference intensity of the complex wavefront is convolved with a comb function resulting from the discrete sampling effect of pixels, which implies that the light intensity *I*_1–3_ and complex wavefront *U*_O_ are discretized. Then, the actual diffractive reconstruction process becomes

(10) $$\begin{array}{l} U_{d}(m\Delta x,n\Delta y)=\mathrm{IDFT}\left\{\mathrm{DFT}\left\{U_{o}(m\Delta x,n\Delta y)\right\}e^{i\frac{2\pi }{\lambda }d\sqrt{1-{\left(\lambda mf_{x0}\right)}^{2}-{\left(\lambda nf_{y0}\right)}^{2}}}\right\},\\ m=-\frac{M}{2},-\frac{M}{2}+1,\ldots \frac{M}{2}-1,\frac{M}{2},n=-\frac{N}{2},-\frac{N}{2}+1,\ldots \frac{N}{2}-1,\frac{N}{2} \end{array}$$

where *M* × *N* is the number of pixels, Δ is the pixel size, *f*_*x*0_ and *f*_*y*0_ are the sampling intervals in the frequency domain. DFT and IDFT denote the discrete Fourier transform and inverse discrete Fourier transform, respectively. This discretization effect leads to errors in the reconstructed wavefront. The steeper the surface is, the greater reconstruction error will be. A simulation is conducted to reveal the reconstruction error resulting from the pixel discretization. The simulated “continuous” wavefront contains 2048 × 2048 pixels with a pixel size of 0.5 μm, and after discretization, the number of pixels is 256 × 256 and the pixel size is 4 μm. In addition, the diffraction distance used for the simulation is 0.05 mm. Figure 2 depicts the relationship between the maximum terrain inclination angle of a rotationally symmetric paraboloid and the resulting mean absolute error (MAE) phase error of the reconstructed amplitude. This implies that sparsifying fringes by tilting the reference mirror can effectively reduce the reconstruction error. In addition, as shown in Figure 3c, when the maximum angle achieves 23°, the height error in the steep region of the edge increases significantly due to the low sampling rate, with each fringe covering about three pixels.

According to the phase *φ*_d_ map associated with the reconstructed wave *U*_d_, the surface topography *h* can be obtained by Equation (11).

(11) $$h(x,y)=\frac{\lambda }{4\pi }\varphi_{d}(x,y)$$

The lateral resolution of measurement is identified as the smaller one between the Rayleigh limit caused by diffraction and the discretization effect of the camera pixels. In this system, the latter is smaller and the determined lateral resolution is about 170 nm. For the vertical resolution along the optical axis, DHM has the resolution at the nanometer scale [27].

If the surface under test is too steep, the diffracted light along the observation direction becomes weak and the phase map is difficult to be restored from the reconstructed wave, which makes the associated area under test unmeasurable [28]. In this case, the object or illumination light needs to be tilted, which is beyond the scope of this paper. We only consider the immeasurability issue arising from the inability of phase unwrapping for over-dense interference fringes. Theoretically, the maximum terrain inclination angle *τ* that can be measured is determined by the numerical aperture (NA) of the microscope objective *τ* = arcsin(NA). For a 20× micro objective with the NA of 0.4, the maximum terrain inclination angle τ is about 23.6°. However, the resulting fringes are dense. The number of pixels *n* occupied by a single fringe is

(12) $$n=\frac{\lambda \times M}{2\times \tan(\tau )\times p}$$

where *λ* is the wavelength of the light source, β is the magnification of the micro objective, *τ* is the relative tilt angle of the object to be measured and the reference mirror, and p is the pixel size. According to Equation (12), a fringe occupies less than 2 pixels in an extreme case with NA = 0.4, β = 20× and *p* = 3.45 μm. By tilting the reference mirror, the fringe can be adjusted to be sparse. For example, when the reference mirror is tilted by 10°, each stripe occupies 4 pixels, which can improve the accuracy of the measurement. In practice, the maximum terrain angle *τ* will be somewhat smaller than the theoretical value due to the low reflectivity of the measured surface.

## 3. Tilted Wave Digital Holographic Microscopy

**Figure 4:**
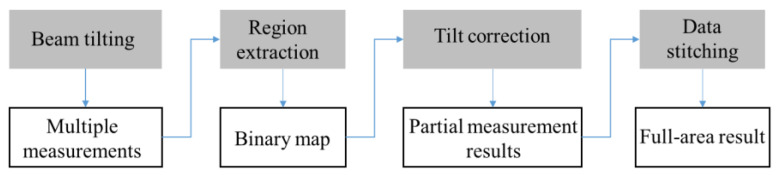
Flowchart of the proposed method for measuring high-slope objects.

The proposed strategy consists of four steps, including beam tilting, region extraction, tilt correction, and data stitching. The main flowchart is depicted in Figure 4.

Firstly, a series of interferograms is obtained by adjusting the tilt angles of the reference beam via tilting the reference mirror or via rotating a pair of prisms, and then the interference fringes associated with different areas of the surface under test are sparsified in sequence. Next, a complex amplitude is obtained from the phase-shifted interferograms and the partial measurable areas with recognizable fringes in each restored phase map are extracted. Then, the object wave is worked out by inverse diffractive reconstruction and tilt correction is performed between adjacent sub-regions. Finally, the sub-regions are stitched together to obtain a full-area measurement of the surface topography.

### 3.1. Beam Tilting

In those areas with high slopes, the resulting interference fringes are too dense to be recognized. By adjusting the reference mirror to different tilt angles or by rotating a pair of prisms, the interference fringes associated with high-slope areas can be sparsified in turn. The tilt direction of the mirror is determined according to the transition zone between the identifiable region and the fringe-dense area. The mirror is tilted slightly towards an arbitrary direction first. If the fringes in the transition zone become dense, the reference mirror is then tilted towards the opposite direction, and vice versa. The reference mirror is mechanically adjusted, and a motored scanning galvanometer can be adopted to achieve automatic and fine adjustment.

With the simultaneous phase shifting system, four interferograms with relative phase shifts of *π/2* can be obtained by a single shot without introducing measurement errors caused by environmental vibrations. Interpolation is then implemented based on the pixels with the same phase shift and eventually four phase-shifted holograms are obtained with all the vacant pixels filled.

### 3.2. Region Extraction

Those measurable regions with sparse fringes are then identified [29,30] in accordance with the total differentiation of the real and imaginary parts of the complex amplitudes. The pixels with large differentiation values are labeled to 1. Otherwise, they are labeled to 0. Mathematical morphology operator ‘open’ is used to eliminate isolated defects.

Then, split the binary measurability map into steep regions and planar regions. A morphology operator ‘close’ with a structuring element of a large size is imposed to connect the points of high steepness. Then, the image is divided into several connected regions. The largest planar region is marked as 1 and the rest are marked as 0. This binarization mask associated with the *k*-th measurement result is denoted as $W^{\left(k\right)}(x,y)$ and the target area can be obtained accordingly.

In practice, the user-set structuring element size for the ‘close’ operation is recommended to be about one-thirtieth of the whole image size. A smaller size cannot make high steepness points to be connected, resulting in a failed segmentation. On the other hand, a larger size will lead to coarse segmentation. Additionally, it can be verified whether the result of region extraction covers the entire area by summing all binarization masks.

Then, a relative phase map between the object surface and the reference plane is obtained via the diffractive reconstruction. Since the position of the sample remains fixed during the whole measurement process, all the resulting complex amplitudes associated with different tilt angles of the reference beam are reconstructed using the same distance. Diffractive reconstruction is implemented from the frame of the hologram with the greatest recognizable area, and all the other holograms are processed using the same diffraction distance. An unwrapping step is performed subsequently using the quality-guided algorithm [31].

### 3.3. Data Stitching

Since the reconstructed object waves are associated with the subtractions between the real object wave and different tilt reference beams, tilt correction is conducted subsequently to unify the measurement results associated with different tilt angles into the same coordinate system.

First, a central region $Z^{\left(0\right)}(x,y)$ is selected as a reference. A plane is fitted to the difference between the overlapped regions associated with each measurement region $Z^{\left(k\right)}(x,y)$ and the base $Z^{\left(0\right)}(x,y)$, namely $\Delta Z^{\left(k\right)}\left(x,y\right)=Z^{\left(k\right)}\left(x,y\right)-Z^{\left(0\right)}(x,y)$. The superscript *k* denotes the *k*-th measurement result.

The objective function is defined as

(13) $$\underset{a_{1}^{\left(k\right)},a_{2}^{\left(k\right)},c^{\left(k\right)}}{\min}\underset{x,y}{\sum }W^{\left(0\right)}(x,y)W^{\left(k\right)}(x,y){\left(a_{1}^{\left(k\right)}x+a_{2}^{\left(k\right)}y+\Delta Z^{\left(k\right)}(x,y)-c^{\left(k\right)}\right)}^{2}$$

where $W^{\left(k\right)}(x,y)$ is the binarization mask defined in Section 3.2. The unknown coefficients $a_{1}^{\left(k\right)}$, $a_{2}^{\left(k\right)}$, $c^{\left(k\right)}$ of the *k*-th plane are obtained accordingly. Next, the measurement results are corrected by subtracting the fitted tilt planes. Theoretically, the topographies from different results should have the same height value after tilt correction. But in practice, there are subtle differences due to noise. Therefore, the topographies in the common areas are averaged to obtain the final measurement result.

## 4. Experimental Verification and Discussion

To demonstrate the performance of the proposed method, a DHM system is established as shown in Figure 2. The details of the components are list in Table 1. The light source is a laser diode (LD) CPS532 by Thorlabs with a central wavelength of 532 nm, and the illumination is optimized using a spatial filter system with a pinhole diameter of 20 μm. The use of a LD instead of a He-Ne laser significantly reduces coherent noise, but it requires more careful alignment [32]. By adjusting the polarizer and the half-wave plate, the light intensity of the reference arm and the object arm can be balanced, thereby obtaining high-contrast interference fringes. Because of the short coherence length in this system, which is 0.14 mm, a Linnik interferometer is constructed by using the same microscope objectives in the object arm and the reference arm, which also reduces the effect of optical aberrations. Polarizing optical components including a polarizer, a half-wave plate, two quarter-wave plates and a polarizing beam splitter help eliminating the secondary reflections arising in the system. Finally, four interferograms with a relative phase shift of π/2 can be recorded on a polarization camera Mako G-508B POL simultaneously. Furthermore, all wave plates used in the experimental system are Thorlabs’ true zero-order wave plates. The part number of the quarter-wave and half-wave plates are WPQ10E-532 and WPH10E-532, respectively. They are fabricated from a liquid crystal polymer (LCP) and can provide stable performance over a range of wavelengths and a large range of angles of incidence.

**Figure 5:**
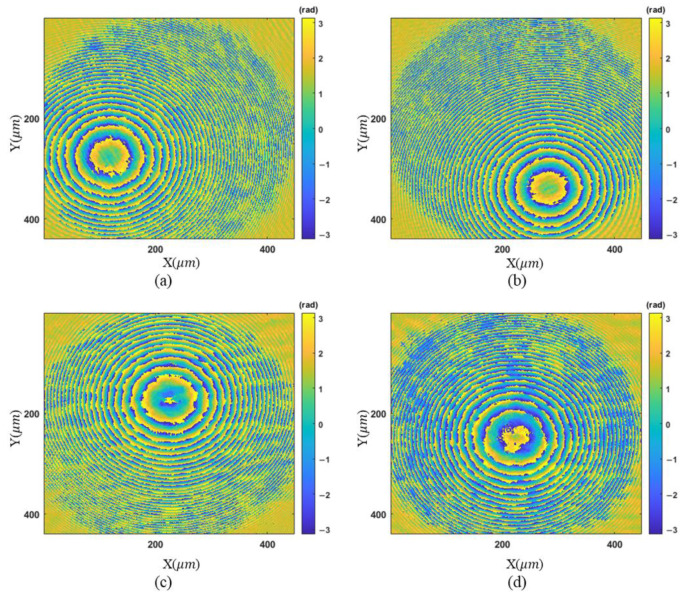
Wrapped phase maps for the convex mirror: (**a**–**d**) phase maps associated with different tilt angles.

Two samples are measured to demonstrate the proposed method. A convex mirror is measured first. Four phase maps associated with different tilt angles are shown in Figure 5. Because of the tilt of the reference mirror, the sparse regions of the fringes are located in different areas.

**Figure 6:**
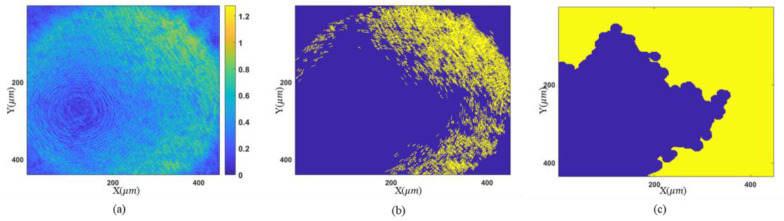
Procedure of region extraction: (**a**) gradient map of complex amplitude; (**b**) binarization map; (**c**) result of region extraction.

**Figure 7:**
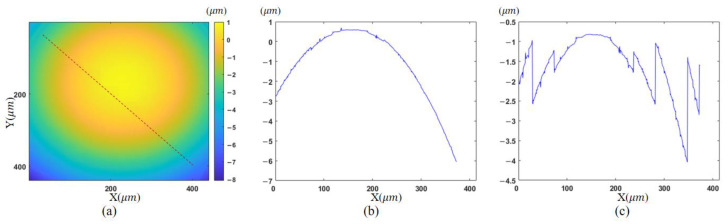
Comparison of the measured results: (**a**) reconstructed topography; (**b**) profile of proposed method; (**c**) profile of Figure 5a.

With the proposed morphology-based segmentation algorithm, measurable regions with sparse fringes are obtained. The complete process of region extraction is given in Figure 6, and the result is illustrated in Figure 6c, where high-slope parts to be discarded are colored yellow. Tilt correction for other measurements is conducted based on Figure 5d, because it has a common area with the others after region extraction. The final height map is presented in Figure 7a. The reference mirror tilted 4.94° from Figure 5d to Figure 5a, according to the tilt correction result, and the fringe density in the target zone becomes 0.31 times the original. This is equivalent to using an objective lens with a magnification of 3.18 times the original one.

To demonstrate the effectiveness of our approach, the final fusion result of multiple reconstructed topographies in Figure 7a is compared with the partial result in Figure 5a. Due to the dense fringes in the upper right area, the correct phase map cannot be obtained by unwrapping in this area. The resulting profile along the diagonal shown in Figure 7c is inaccurate. With the developed algorithm, the whole profile at the same location is extracted in the fused result, as depicted in Figure 7b.

**Figure 8:**
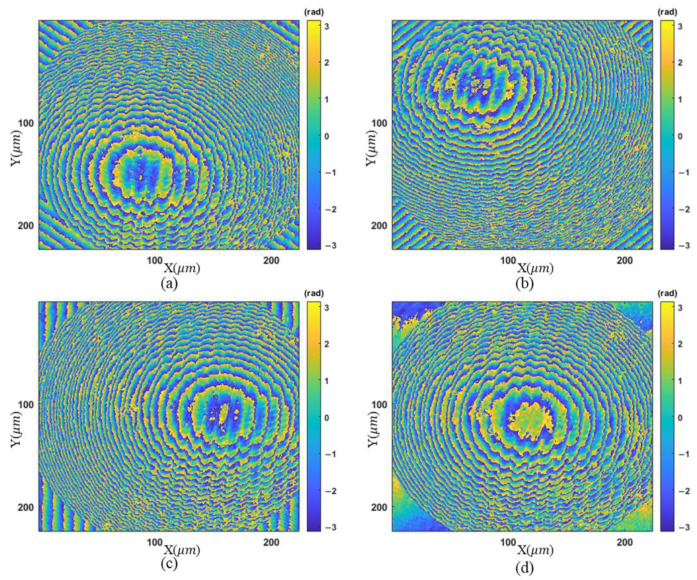
Wrapped phase maps for the concave surface: (**a**–**d**) phase maps associated with different tilt angles.

**Figure 9:**
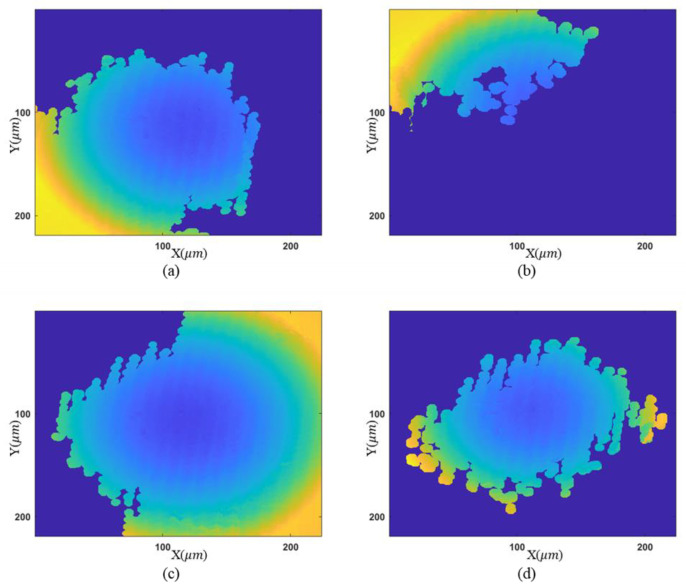
Unwrapped phase maps: (**a**–**d**) phase maps associated with different tilt angles.

**Figure 10:**
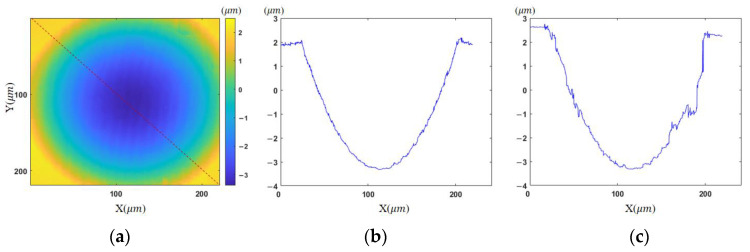
Reconstruction results for the concave surface: (**a**) fused topography; (**b**) profile of proposed method; (**c**) profile of Figure 8a.

Another concave micro-lens mold is then measured. The wrapped phase maps and unwrapped phase maps after region extraction are shown in Figure 8 and Figure 9, respectively. The stitched topography is illustrated in Figure 10a.

For comparison, the profiles along the diagonal are given in Figure 10b,c, corresponding to the final result and Figure 8a, respectively. There are some regions in Figure 8a that cannot be unwrapped correctly and the complete measurement is obtained using the proposed algorithm. The fringe density becomes 0.42 times the original from Figure 8d to Figure 8a in the lower left zone. It is equivalent to using an objective lens with a magnification of 2.37 times the initial one. Due to the low reflectivity, these two samples cannot be measured reliably using the commonly adopted coherent scanning interferometry. Comparable validation by other instruments will be conducted in the future.

**Figure 11:**
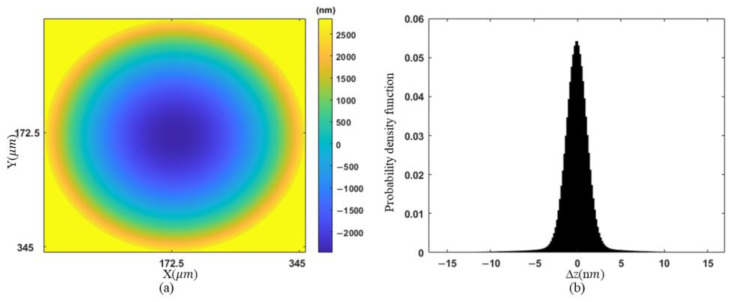
Uncertainty analysis of measurements: (**a**) simulated rotationally symmetric paraboloid; (**b**) the probability density function of ∆z.

In addition, an uncertainty analysis of measurements is conducted. The error sources of DHM include the shift of central wavelength of light source, polarization direction error and transmittance variation in the polarization image sensor, and the error of the diffractive distance. Monte Carlo simulation is applied for uncertainty analysis. First, a rotationally symmetric paraboloid shown in Figure 11a is generated as an object, which is of radius of curvature 176.64 μm and height 5.08 μm. Then, we generate the hologram and reconstruct the topography by adding the major error factors to the simulation, where the central wavelength of the light source is uniformly distributed from 531 to 533 nm, the diffraction distance is normally distributed with a standard deviation of 1/10 of the theoretical value, the tangent value of the extinction ratio is uniformly distributed from 500 to infinity, and the angle of the micro-polarizer is uniformly distributed within an error range of ±1.5°. In addition, 40 dB of noise and 12 bits of quantization error are added to the sensor. The simulation process is looped 1000 times and the output distribution of reconstruction error Δz is shown in Figure 11b. The uncertainty of reconstruction height is 3.5 nm with 95% coverage probability, which is in an acceptable range.

## 5. Conclusions

In general, a measuring technique with high flexibility and low cost is proposed for high-slope micro-nano structures. As a DHM technique, it can reveal object surface with vertical resolution at the nanometer scale and with the same lateral resolution as a normal microscope objective. A series of holograms are recorded by tilting the reference mirror to sparsify the interference fringes in the high-slope regions. With this technique, we can obtain the complete surface topography with high-slope surfaces without the use of more advanced wave-front modulation elements such as SLM or deformable mirrors. This technique may find potential applications in various fields such as biology, materials science, and microelectronics.

## Figures and Tables

**Table 1:** Specifications of components in the system.

Number	Component	Specification
1	Laser diode	532 nm, 4.5 mW
2	O. L	Olympus MPLFLN20
3	pinhole	Ø20 ± 2 µm
4	Lens1	Ø25.4 mm, f = 125.0 mm, 400–700 nm
5	Polarizer	Ø25.4 mm, 400–700 nm
6	1/2 W. P	Ø25.4 mm, 532 nm
7	Lens2	Ø25.4 mm, f = 300.0 mm
8	P. B. S	25.4 × 25.4 × 25.4 mm; 420–680 nm
9	1/4 W. P	Ø25.4 mm, 532 nm
10	Mirror	Ø25.4 mm, 400–750 nm
11	T. L	f = 180 mm, 350–700 nm
12	Polarization camera	Mako G-508B POL, resolution: 2464 × 2056; pixel size: 3.45 µm × 3.45 µm

## Data Availability

Data are contained within the article.
